# A phase I study of afatinib for patients aged 75 or older with advanced non-small cell lung cancer harboring EGFR mutations

**DOI:** 10.1007/s12032-018-1098-3

**Published:** 2018-02-08

**Authors:** Hisashi Tanaka, Kageaki Taima, Yoshihito Tanaka, Masamichi Itoga, Yoshiko Ishioka, Hideyuki Nakagawa, Keisuke Baba, Yukihiro Hasegawa, Shingo Takanashi, Sadatomo Tasaka

**Affiliations:** 1 Department of Respiratory Medicine, Hirosaki University Graduate School of Medicine, 5, Zaifu-cho, Hirosaki, 036-8562 Japan; 2 Department of Respiratory Medicine, Hirosaki National Hospital, 1, Tomino, Hirosaki, Japan; 3 Department of Respiratory Medicine, Aomori Prefectural Central Hospital, 1-1-2, Higashitukurimichi, Aomori, Japan; 4 Health Administration Center, Hirosaki University, 5, Zaifu-cho, Hirosaki, Japan

**Keywords:** Non-small cell lung cancer, Epidermal growth factor receptor mutation, Afatinib, Elderly patients

## Abstract

This phase I trial was conducted to determine the maximum tolerated dose (MTD) and recommended dose of afatinib for phase II trial in elderly patients with advanced non-small cell lung cancer (NSCLC) harboring epidermal growth factor receptor (EGFR) mutations. The study used a standard 3 + 3 dose escalation design. Patients aged 75 years or older with advanced NSCLC harboring EGFR mutations were enrolled. The doses of afatinib, which were given once daily, were planned as follows: level 1, 20 mg/day; level 2, 30 mg/day; level 3, 40 mg/day. Dose-limiting toxicity (DLT) was defined as grade 4 hematologic, persistent grade > 2 diarrhea for > 2 days despite concomitant medications or grade 3 non-hematologic toxicity. DLT was evaluated during day 1–28. Fifteen patients were enrolled. Patient characteristics were: male/female 3/12; median age 79 (range 75–87); PS 0/1, 2/13. Six patients have been treated at levels 1 and 3, and three patients at level 2. At level 1, one of six patients experienced grade 3 rush, grade 3 anorexia, and grade 3 infection as DLTs. At level 2, none of three patients experienced a DLT. At level 3, two patients developed grade 3 diarrhea, one of whom also experienced grade 3 anorexia. Most frequent adverse events of any grade were diarrhea, paronychia, rush, and nausea. Most patients at level 2 and 3 required dose reduction in 3 months. MTD was defined as 40 mg/day, and recommended dose for phase II study in elderly patients was 30 mg/day.

## Introduction

Lung cancer is the leading cause of cancer-related death in the world, with non-small cell lung cancer (NSCLC) accounting for 85% [1]. Along with an aging society, elderly patients diagnosed with lung cancer also have been increasing. The rate of the patients aged 80 years or older is 14%, and those aged patients 70 years or older was 47% of all lung cancers [2]. Therefore, it has become increasingly important to establish more effective and safe treatment for elderly patients. Patients with epidermal growth factor receptor (EGFR) mutations are recommended to receive molecular target therapy such as gefitinib, erlotinib and afatinib [3–7]. More recently, osimertinib which is the third generation EGFR-tyrosine kinase inhibitor (TKI), is used for the patients with T790 M-positive NSCLC [8]. Afatinib is a novel, potent, small-molecule ErbB family blocker that covalently binds and irreversibly blocks signaling through activated EGFR, human epidermal growth factor receptor 2 (HER2), and ErbB4 receptors [9, 10]. In two pivotal phase III studies, afatinib showed better progression-free survival (PFS) than standard platinum-based chemotherapy [7, 11]. In addition, LUX-Lung 7 study revealed that afatinib might offer improved PFS compared with gefitinib and that the number of patients discontinuing treatment due to adverse events (AEs) was similar in each group [12]. Diarrhea and skin rush were the commonest AEs for afatinib. Moreover, grade > 3 AEs were more frequent for afatinib (31%) than gefitinib (19.5%) [12]. Thus, the choice of TKI should be based on the balance between efficacy and safety because the toxicity profiles are different among TKIs. Regarding as the definition of elderly patients, we have searched through the published literature. The elderly are considered as > 75 years in Europe, whereas > 65 in USA [13, 14]. In the Japanese lung cancer guideline, elderly patients are defined as > 75 years of age and gefitinib is recommended for the treatment of mutation-positive NSCLC in such patients. Two prospective phase II studies of gefitinib that recruited patients with NSCLC harboring EGFR mutation who were ineligible for cytotoxic chemotherapy owing to their PS or age were reported [15, 16]. In addition, a prospective randomized phase II study that compared erlotinib to vinorelbine for the patients aged 70 years or older was reported from Taiwan [17]. However, there has been no prospective study that examines the efficacy and safety of afatinib in elderly patients yet. Therefore, we conducted a phase I study to determine the maximum tolerated dose (MTD) and the recommended dose (RD) of afatinib for phase II trial in elderly patients aged 75 or older with NSCLC harboring EGFR mutations.

## Materials and methods

### Study design

This clinical trial was an open-label, multicenter trial utilizing a standard 3 + 3 dose escalation protocol involving 3 institutions in Aomori prefecture, Japan. Patients received afatinib once daily with following doses: level 1, 20 mg/day; level 2, 30 mg/day; level 3, 40 mg/day. Level 0 is an option for the patient who requires dose reduction due to the toxicities in level 1. AEs were evaluated by Common Terminology Criteria for Adverse Events (CTCAE). Dose-limiting toxicity (DLT) was defined as grade > 3 non-hematologic toxicity; persistent grade > 2 diarrhea for > 2 days, despite concomitant medications; grade > 4 hematologic toxicity; febrile neutropenia; any grade interstitial lung disease. DLTs were evaluated during first 28 days to determine MTD. The MTD was defined as the highest dose at which the incidence of DLTs was less than or equal to 33.3%. The RD of afatinib for phase II study was defined as one level lower than MTD. Changes in the dose and the number of enrolled patients in the next dosage group were determined by the toxicity. In this protocol, if a DLT was not observed in any of the first three patients, the dose was escalated to the next level. If a DLT was occurred in one of three patients, three additional patients were recruited at the same dose. If a DLT occurred in only one of six patients, dose escalation was permitted. If DLTs were observed in two of six or more patients, dose escalation was not permitted. This level was defined as the MTD. If a DLT occurred in only one of six or fewer patients in level 3, we were not able to define the MTD, and the RD was regarded as 40 mg/day. Treatment was discontinued when the patients had disease progression, unacceptable toxicity was observed, or the patients refused treatment. This study was performed in accordance with the principles of the Declaration of Helsinki and Good Clinical Practice guidelines. This study was approved by the institutional review boards of Hirosaki University, Hirosaki National Hospital, and Aomori Prefectural Central Hospital. Patients decided participation in this trial after detailed explanation; written informed consent was obtained from all patients before the entry. This study was registered with the University Hospital Medical Information Network (UMIN). Clinical trial number UMIN000016441.

### Eligibility criteria

Eligible patients were as follows: ≧ 75 years, histologically or cytologically confirmed NSCLC, EGFR mutations. The patients had measurable disease as defined by the Response Evaluation Criteria in Solid Tumors (RECIST) (version 1.1), an Eastern Cooperative Oncology Group (ECOG) performance status (PS) 0–1. Patients also had adequate organ function; neutrophil count ≧ 1500/mm^3^, hemoglobin ≧ 9.0 g/dL, and platelet count ≧ 100,000/mm^3^, aspartate transaminase and alanine transaminase levels ≦ 100 IU/L, creatinine ≦ 1.5 mg/dl and creatinine clearance≧ 40, total bilirubin concentration ≦ 1.5 mg/dl, and PaO2 ≧  60 Torr or SpO2 ≧ 95%. The life expectancy more than 12 weeks was required. Patients who had undergone thoracic radiation therapy were required to finish their last treatment at least 12 weeks prior to the enrollment in the protocol. The patients were required to finish their prior chemotherapy at least 4 weeks before the enrollment. Patients with symptomatic central nervous system metastasis, uncontrolled pleural effusion requiring drainage treatment, the use of corticosteroid, hypersensitivity to afatinib or the use of immunosuppressive drugs, or concomitant disease such as active peptic ulcer, heart disease, interstitial pneumonia or pulmonary fibrosis, mental disorder, cerebrovascular disease, infection, and diabetes mellitus were excluded.

### Evaluation and statistical analysis

The primary endpoints were the MTD and the RD of afatinib for phase II trial in elderly patients with advanced NSCLC harboring EGFR mutations. The overall response rate (ORR) and the PFS were secondary endpoints. The PFS was estimated using the Kaplan–Meier method. The PFS has been defined as the time from the date of treatment initiation to the date of disease progression, death, or the last contact. If neither event is observed, it is considered to be censored with the latest observation date. If the date on which disease progression is confirmed has exceeded 8 weeks after the last examination, it shall be censored with the previous examination date. If post-treatment is started, it is considered to be censored with the date of initiation. If the event is unknown in the case of transfer or non-arrival, it will be censored with the final date when the patient survival was confirmed. Statistical analyses were performed using JMP 10 (SAS Institute, Cary, NC, USA). Tumor responses were assessed using chest radiography and computed tomography scan at every 8 weeks until disease progression. Unidirectional measurements were adopted on the basis of the RECIST, version 1.1.

## Results

### Patient characteristics

From February 2015 to September 2016, a total of 15 patients were enrolled from three participating institutions in Aomori, Japan. Table 1 shows the characteristics of the 15 eligible patients. Six patients were included in the afatinib 20 mg/day cohort, three patients were in the 30 mg/day cohort and six patients were in the 40 mg/day cohort. Six patients were included in each of the 20 mg/day and the 40 mg/day cohort because one of the first three patients experienced a DLT, requiring the enrollment of additional three patients. Three male (20%) patients and 12 female (80%) patients, with a median age of 79 years (range, 75–87 years), were included. The histology was adenocarcinoma in all patients. Two patients had stage IIIB, 11 had stage IV, and two had recurrent disease. The EGFR mutation status was as follows: exon 19 del/exon 21 L858R/exon 18 G719X in 3/11/1, respectively. Two patients (13.3%) received prior chemotherapy (one patient received gefitinib as the first-line treatment and erlotinib as the second-line treatment, and the other received platinum-based chemotherapy), and 13 patients (86.7%) were chemo-naïve.

**Table 1:** Patient characteristics (*N* = 15)

Gender	
Male/female (*n*)	3/12
Age (years), median (range) 79 (75–87)	
ECOG PS [*n* (%)]	
0–1	13 (86.7)
2	2 (13.3)
Clinical stage [*n* (%)]	
IIIB	2 (13.3)
IV	11 (73.4)
Recurrence	2 (13.3)
Histological type [*n* (%)]	
Adenocarcinoma	15 (100)
Smoking history [*n* (%)]	
Never smoker	12 (80)
Light smoker	3 (20)
EGFR mutation [*n* (%)]	
Exon 19 del	3 (20)
Exon 21 L858R	11 (73.4)
Exon 18 G719	1 (6.6)
No. of prior treatment regimen [*n* (%)]	
0	13 (86.7)
1–2	2 (13.3)

*ECOG* Eastern Cooperative Oncology Group; *PS* performance status

### MTD and toxicity analysis

The DLTs observed during day 1–28 at each dose level are summarized in Table 2. Six patients were enrolled in the 20 mg/day cohort, three patients in the 30 mg/day cohort, and six patients in the 40 mg/day cohort. One patient experienced DLTs at the 20 mg/day cohort. Grade 3 rash, anorexia and infection were observed in a 77-year-old woman. At the 30 mg/day cohort, no DLT was observed in 3 patients. At the 40 mg/day cohort, two patients experienced DLTs. Grade 3 diarrhea was observed in a 75-year-old woman, grade 3 diarrhea and anorexia were observed in an 80 year-old woman. The frequency of DLTs was 33.3% at the 40 mg/day cohort. We considered that the MTD was 40 mg/day. Eight of nine patients (88.8%) enrolled in 30 mg/day and 40 mg/day cohorts, required dose reduction of afatinib within 3 months. The primary reasons for dose reduction included diarrhea, nausea, skin-related AEs, and mucositis. The major toxicities in all term are given in Table 3. No grade 3 or higher hematologic toxicities were observed at any level. Grade 3 non-hematologic toxicities were skin rush (20%), anorexia (13.3%), diarrhea (13.3%), and nausea or vomiting (6.6%). Most of low-grade non-hematologic toxicities were nausea, vomiting, anorexia, fatigue, paronychia, oral mucositis, and infection, which were generally mild and reversible. There were no treatment-related deaths in this phase I study.

**Table 2:** Observed dose-limiting toxicities in treatment during day 1–28 at each dose level

Treatment level	Afatinib (mg/day)		
	Level 1 20 mg/day *n* = 6	Level 2 30 mg/day *n* = 3	Level 3 40 mg/day *n* = 6
Patients number	6	3	6
DLT, n (%)	1 (16.7)	0	2 (33.3)
Details of DLTs	77 years, female Grade 3 rash, anorexia and infection		75 years, female Grade 3 diarrhea 80 years, female Grade 3 diarrhea, and anorexia

*DLT* dose-limiting toxicities

**Table 3:** Toxicity in patients treated with afatinib (*n* = 15)

Afatinib	20 mg		30 mg		40 mg		All dose	
	*n* = 6		*n* = 3		*n* = 6		*n* = 15	
Grade (CTCAE) ver4.0	1–2	3	1–2	3	1–2	3	All grade (%)	3 (%)
Neutropenia	1	0	0	0	0	0	1 (6.6)	0
Anemia	3	0	3	0	1	0	7 (46.6)	0
Thrombocytopenia	0	0	0	0	0	0	0	0
Nausea, vomiting	3	0	1	0	2	1	7 (46.6)	1 (6.6)
Anorexia	1	1	2	0	2	1	7 (46.6)	2 (13.3)
Fatigue	2	0	2	0	2	0	6 (40)	0
Diarrhea	6	0	3	0	4	2	15 (100)	2 (13.3)
Skin rush	2	1	3	0	4	2	12 (80)	3 (20)
Paronychia	0	0	3	0	3	0	6 (40)	0
Oral mucositis	0	0	2	0	3	0	5 (33.3)	0
Increased AST	2	0	0	0	1	0	3 (20)	0
Infection	1	1	0	0	1	0	3 (20)	0
Increased Creatinine	0	0	0	0	1	0	1 (6.6)	0

*AST* Aspartate aminotransferase; *CTCAE* common terminology criteria for adverse events

### Efficacy

**Fig. 1:**
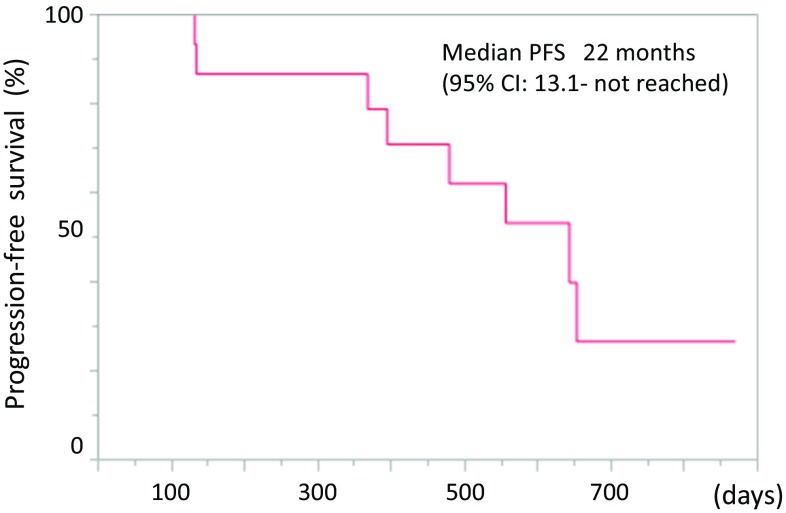
Kaplan–Meier analysis of progression-free survival for all 15 treated patients

The response to afatinib in the intent-to-treat population is given in Table 4. Eleven patients attained a partial response (PR), but no patients attained a complete response (CR). The ORR was 73.3%, and three patients (20.1%) had stable disease (SD). The median follow-up time at analysis (August 2017) was 568 days. Eight patients (53.3%) had PD, the median PFS was 22 months (95% CI, 13.1—not reached) (Fig. 1).

**Table 4:** Response to afatinib in the intent-to-treat population

Response	Number of patients	%
Complete response	0	0
Partial response	11	73.3
Stable disease	3	20.1
Progressive disease	0	0
Not evaluable	1	6.6
Response rate	73.3%

## Discussion

This is the first phase I study demonstrating that afatinib has manageable safety profiles for patients aged 75 or older with advanced NSCLC harboring EGFR mutations. The MTD was defined as 40 mg/day, and the RD for phase II study in elderly patients was 30 mg/day. The MTD-defining DLTs were rash, anorexia, infection, and diarrhea, which recovered by the temporal discontinuation or dose reduction. In a previous phase I study of afatinib in patients with advanced solid tumors, median age of the patients was 56 years, and the RD for phase II study was reported as 50 mg/day [18]. Similarly, a phase I study in younger patients with previously treated NSCLC showed that 50 mg/day of afatinib was well tolerated [19]. Thus, the starting dose of afatinib was 40 mg/day in a phase III NSCLC trial [20]. The most frequent drug-related AEs were diarrhea, dry skin, stomatitis, rash, paronychia and anorexia, and an increased frequency and severity of drug-related AEs were observed accompanied with higher doses of afatinib [18, 19]. In LUX-lung 7, Paz-Ares and colleagues reported that there was no difference of overall survival (OS) among patient subgroups, and similar median OS was seen at cutoffs of 60, 65, 70, and 75 years [21]. In that study, however, the number of patients aged 75 or older were small, and moreover, AEs in this age group was not reported. The efficacy and safety of afatinib in such patients has not been clear yet.

Previous reports suggested that dosing of small-molecule TKIs according to body surface area (BSA) might be meaningful. In a phase I trial of sunitinib, low BSA was associated with higher incidence of severe AEs and DLTs [22]. In the first-generation EGFR-TKIs (gefitinib and erlotinib), dose reduction was not required according to BSA [23]. Recently, Wada and colleagues reported that lower BSA (< 1.50 m^2^) was significantly associated with higher frequency of diarrhea grade > 2 in patients treated with afatinib [24]. In our study, median BSA of recruited patients was 1.35 m^2^ (range 0.93–1.59), comparable with the subject of a previous study [24]. We observed higher frequency of diarrhea than in a previous report, which might be due to older age of the study subjects. In our study, mean BSA of the patients who experienced DLTs was 1.23 m^2^, although the sample size was too small to discuss the relationship between low BSA and DLTs in afatinib treatment.

The efficacy was not the primary end point of this study, but the ORR in our study was 73.3%, similar to the results of the previously reported clinical trials [7, 20, 21].

In conclusion, the MTD was defined as 40 mg/day, and the RD for phase II study in elderly patients aged 75 or older was 30 mg/day. Afatinib showed a manageable safety profile and efficacy comparable with those described in the previous studies, although they remain to be evaluated in a phase II study. Now a phase II clinical trial that evaluates the efficacy of afatinib in elderly patients aged 75 or older is ongoing in Japan (UMIN000017877).

## Abbreviations

MTD: Maximum tolerated dose
NSCLC: Non-small cell lung cancer
EGFR: Epidermal growth factor receptor
ECOG: Eastern Cooperative Oncology Group
PS: Performance status
DLT: Dose-limiting toxicity
AE: Adverse event
TKI: Tyrosine kinase inhibitor
HER 2: Human epidermal growth factor receptor 2
PFS: Progression-free survival
DLT: Dose-limiting toxicity
RD: Recommend dose
UMIN: University Hospital Medical Information Network
RECIST: Response evaluation criteria in solid tumors
CTCAE: Common terminology criteria for adverse events
ORR: Overall response rate
PR: Partial response
CR: Complete response
SD: Stable disease
OS: Overall survival
